# Decision making based on hybrid modeling approach applied to cellulose acetate based historical films conservation

**DOI:** 10.1038/s41598-021-95373-0

**Published:** 2021-08-09

**Authors:** Abeer Al Mohtar, Moisés L. Pinto, Artur Neves, Sofia Nunes, Daniele Zappi, Gabriele Varani, Ana Maria Ramos, Maria João Melo, Nadja Wallaszkovits, Juan Ignacio Lahoz Rodrigo, Kerstin Herlt, João Lopes

**Affiliations:** 1 CERENA, Departamento de Engenharia Química, Instituto Superior Técnico, Universidade de Lisboa, 1049-001 Lisbon, Portugal; 2 iMed.ULisboa, Faculdade de Farmácia, Universidade de Lisboa, Av. Prof. Gama Pinto, 1649-003 Lisbon, Portugal; 3 LAQV-REQUIMTE, Departamento de Conservação e Restauro and Departamento de Química, NOVA School of Science and Technology, Universidade NOVA de Lisboa, 2829-516 Caparica, Portugal; 4 Biosensor, Via degli Olmetti 44, 00060 Formello, RM Italy; 5 Phonogrammarchiv of the Austrian Academy of Sciences, Liebiggasse 5, 1010 Vienna, Austria; 6 Filmoteca Valenciana, Institut Valencià de Cultura, Plaça de l’Ajuntament 17, 46002 Valencia, Spain; 7 Deutsches Filminstitut & Filmmuseum Schaumainkai 41, 60596 Frankfurt, Germany

**Keywords:** Chemistry, Engineering, Materials science

## Abstract

Preserving culture heritage cellulose acetate-based historical films is a challenge due to the long-term instability of these complex materials and a lack of prediction models that can guide conservation strategies for each particular film. In this work, a cellulose acetate degradation model is proposed as the basis for the selection of appropriate strategies for storage and conservation for each specimen, considering its specific information. Due to the formulation complexity and diversity of cellulose acetate-based films produced over the decades, we hereby propose a hybrid modeling approach to describe the films degradation process. The problem is addressed by a hybrid model that uses as a backbone a first-principles based model to describe the degradation kinetics of the pure cellulose diacetate polymer. The mechanistic model was successfully adapted to fit experimental data from accelerated aging of plasticized films. The hybrid model considers then the specificity of each historical film via the development of two chemometric models. These models resource on gas release data, namely acetic acid, and descriptors of the films (manufacturing date, AD-strip value and film type) to assess the current polymer degradation state and estimate the increase in the degradation rate. These estimations are then conjugated with storage conditions (e.g., temperature and relative humidity, presence of adsorbent in the film’s box) and used to feed the mechanistic model to provide the required time degradation simulations. The developed chemometric models provided predictions with accuracy more than 87%. We have found that the storage archive as well as the manufacturing company are not determining factors for conservation but rather the manufacturing date, off gas data as well as the film type. In summary, this hybrid modeling was able to develop a practical tool for conservators to assess films conservation state and to design storage and conservation policies that are best suited for each cultural heritage film.

## Introduction

Hybrid modeling has become an attractive and sometimes necessary resource in many fields, especially in engineering^1,2^, applied physics^3,4^, environmental and atmospheric sciences^5^. The concept of hybrid modeling may be applied to any type of different models’ conjugation. However, the historical hybrid modeling approach is very often used to describe models that have a component based on first principles and another relying on a data-driven approach. The idea is to take advantage of both worlds’ benefits: the backbone structure of a reliable first principle derived model (e.g., from mass/energy balances) and an empirical component describing relationships that are unknown often due to systems complexity. Examples of the latter are the description of system kinetics or complex relationships. Experimental data are required to estimate the empirical component. This empirical component can be used prior or embedded in the first principles model backbone. In this work we apply hybrid modeling in the conservation of the culture heritage.

A significant percentage of the recent cultural heritage items is found in motion movies made of cellulose acetate, where the worldwide estimation of such holdings within professional film archives is around 18 million of film reels on cellulose acetate^6^. This polymer, first used in the form of cellulose diacetate (CDA) and later on as cellulose triacetate (CTA) as being less prone to degradation, was initially thought of as a very stable material as a whole, noted normally by CA solely. It replaced the inflammable cellulose nitrate, the first thermoplastic material to be used as flexible support for projection image and photographic negatives^7^. However soon the “vinegar syndrome” was reported^8,9^. It originates from the vinegar odor of the acetic acid (AA) released during the degradation process. AA plays a substantial role in accelerating the degradation process because it is a reaction product and catalyst^10^. Nowadays, more than 75 years of visual and audio memories are in danger of total loss. Some studies have been performed aiming at estimating the activation energy of the cellulose acetate (CA) degradation reaction^11^. Other studies gave a rough estimation of the lifetime of CA films based on temperature (T) and relative humidity (RH) conditions^12^. However, these studies did not take into account the crucial autocatalytic behavior of the deacetylation process. A more recent study was performed to determine the onset of the vinegar syndrome but it did not consider the volatility of acetic acid^13^. Neither of the studies provided explicit dependence of degradation evolution as function of all the involved degradation parameters. Moreover, none of these previous works so far accounted for the specificity (film type (black and white, color or sound), manufacturing date, AA off-gassing amount) of each film. Having the ability to predict the state evolution, of each film as function of storage conditions could provide an extremely powerful tool to design a personalized predictive maintenance action plan. To fill this gap, we developed a hybrid model within the framework of the NEMOSINE European project^14^. This project aims at improving the traditional storage solutions, such as freeze storage (below 5 °C), by developing an innovative package with the main goal of energy saving and extent conservation time. One of the main pillars of this innovative solution is the creation of models that simulate the object degradation from gas sensors signals enclosed in the films boxes and other easily accessed films meta data. The proposed solution is of particular interest since the degradation state and its evolution is determined based on off-gas data measured by sensors in real-time. Thus, this solution does not require any physical handling of the films nor spectroscopic or other expensive/inaccessible equipment.

The hybrid model consists in a first-principles model coupled with two multivariate analysis models. The inputs and outputs of the models are indicated in Table 1. In a first stage, a robust mechanistic model (MM) describing the degradation kinetics of the CA polymer with plasticizers is developed, based from on our recent model that describes pure CDA^10^. The degradation state in this work is represented by the cellulose acetate degree of substitution (DS), which is the number of acetyl groups per anhydroglucose ring of the polymer repeating unit. The initial DS represents the 100% nondegraded state of the polymer. The inputs needed for the MM are the initial DS and the simulation time span along with the storage conditions (T, RH, storage box volume, initial concentration of AA in the atmosphere). The output of the MM is the degradation state evolution of the CA polymer as function of time. In the present work, the MM^10^ was adapted to describe the degradation of plasticized CA films, which represent much better the polymer layer that is used as support of the image, through the introduction of a parameter called kinetic correction factor (${k}_{\mathrm{CF}}$). This factor lumps the effect of plasticizer in the case of plasticized films and accounts for additives^15,16^ and film type in case of historical cinematographic films. In the case of plasticized films, this factor is determined upon obtaining the best fit between the adapted MM output and the experimental data of accelerated aging of plasticized films. In the case of historical films, the ${k}_{\mathrm{CF}}$ is the output of the first chemometric model, CM-${k}_{\mathrm{CF}}$, with the manufacturing date and film type as inputs.

**Table 1:** Inputs and outputs of the developed models that constitute the hybrid model to predict the evolution of historical films.

Model	Input	Output
MM model	Initial DS, time span, storage conditions (T, RH, mass of polymer, V box, initial AA in the atmosphere)	Degradation state evolution of pure polymer as function of time
Adapted MM model	Initial DS, time span, storage conditions (T, RH, mass of polymer, V box, initial AA in the atmosphere), ${k}_{\mathrm{CF}}$	Degradation state evolution of plasticized polymer as function of time
CM-${k}_{\mathrm{CF}}$	Manufacturing date, film type (BW, color, sound)	${k}_{\mathrm{CF}}$; correction constant to describe how much faster the film is degrading than the pure polymer
CM-DS	Manufacturing date, off-gas data (rate of AA release, maximum concentration), AD-strip value	DS; estimation of the current DS of the film
Hybrid model	${k}_{\mathrm{CF}}$=output of CM-${k}_{\mathrm{CF}}$, DS = output of CM-DS, time span, storage conditions	Degradation state evolution of the movie film as function of time
Hybrid model—adsorbent functionality	Mass and Henry’s constant (${K}_{{H}_{ad}}$) of the AA adsorbent, AA adsorbent replacement time	Effect of AA adsorbent on the degradation evolution

To determine the state evolution of a film, the starting point (i.e. the current DS) needs to be determined. The current state of degradation could be assessed using spectroscopic methods, like Attenuated Total Reflectance or micro Fourier Transform Infrared spectroscopies (ATR-FTIR or µFTIR, respectively), via a simple linear model^17,18^. In the absence of such resources, which is a common situation in the archives, CM-DS, a second chemometric model is developed to predict the current DS of the film. The inputs for the CM-DS model are the manufacturing date, off-gas data (rate of AA release, and maximum concentration) and AD-strip value. The output is the current DS of the historical film. The CM-${k}_{\mathrm{CF}}$ and CM-DS are coupled to the adapted MM to form the hybrid model that predicts the future historical film degradation evolution as function of storage conditions.

AA vapors are known to be very harmful to culture heritage items^19,20^, where several materials can suffer fast alteration by being exposed to it. In addition to its catalytic role in the degradation process of CA, this noxious volatile acid attacks a variety of artwork materials like lead and lead alloys^21^, copper alloys^22^, paper^23^, shells materials or other calcareous materials. A number of adsorbent materials to purify the atmosphere from AA were studied aiming to slow down the degradation process. The studied adsorbents were mainly zeolites and activated carbons^21^, and more recently Metal Organic Frameworks^24^. For that reason, the effect of the presence of an AA adsorbent is integrated as a functionality in the hybrid model. The user can simulate the effect of the adsorbent amount (${m}_{ad}$), the adsorbent properties (with its Henry’s coefficient; ${K}_{{H}_{ad}}$), as well as the replacement frequency.

**Figure 1:**
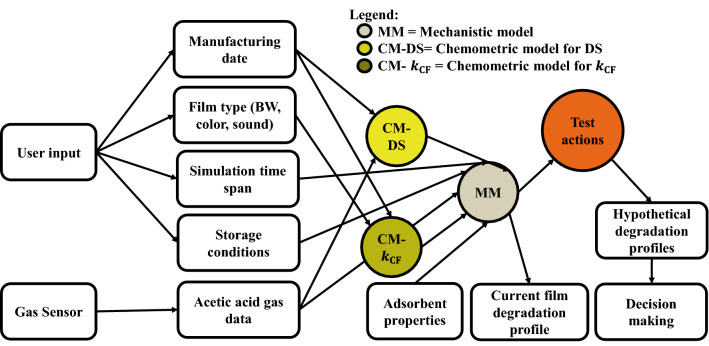
Scheme for the CA based films maintenance decision making tool.

A flow diagram of the developed hybrid model is shown in Fig. 1. The whole package solution enables the user to monitor the current state of the film and to predict its evolution based on off-gas data and user input information. Different scenarios can be considered upon varying the parameters that are impacting the degradation kinetics (e.g. temperature, box volume, adsorbent mass and type, etc.) and estimate the film’s behavior accordingly thus take informative decisions for the best actions.

## Materials and methods

### Preparation of plasticized CDA polymer films

Cellulose diacetate (CDA) films were prepared by dissolving 1 g of the polymer (39.7 wt% acetyl content, *ρ* = 1.3 g/cm^3^, Aldrich) in 100 mL of a mixture 1:1 chloroform (HPLC grade, Aldrich) and acetone (p.a. Aldrich, 99.8%) respectively, inside Erlenmeyer flasks. The polymer solutions were maintained under stirring for 24 h. Then, the plasticizer triphenyl phosphate (TPP, Sigma-Aldrich, ≥ 99%) was added with a concentration of 20 wt% of the polymer. Diethyl phthalate (DEP, Alfa Aesar, 99%) was added with the same concentration to another solution. In both cases the plasticizer was left 24 h to dissolve and after the solutions were poured into individual glassware with a flat base (such as Petri dishes). The solvents were left to evaporate in a desiccator and the films were considered dried, without solvent residues upon reaching constant weight. They were also kept in a desiccator until testing. The films thickness is around 50 μm, were cut in 4 cm x 4 cm pieces and placed in glass supports.

The plasticizers TPP and DEP were chosen because they are representative of the two families of compounds more used on the same periods in motion movies and photography films, phosphates and phthalates respectively^25^.

### Accelerated aging experiments

The CDA films with TPP and DEP plasticizers were aged inside different desiccators with a solution of acetic acid (AA), placed in an oven at 70 °C. The AA solutions were prepared with a concentration of 2% (wt), following the procedure proposed by Cruz et al.^21^. This led to an initial concentration of AA in the vapor phase^10^ of 1.7899 × 10^−5^ mmol/cm^3^. The total mass of the CDA films was circa 0.4 g in each case, placed in a chamber/desiccator with volume 5900 cm^3^ in the case of TPP plasticized films and volume 2550 cm^3^ in the case of DEP plasticized films. The aging experiments were followed by weekly collecting two different samples of each film. Four different micro samples were picked from each film, to be analyzed by µFTIR, in a total of eight spectra for each time considered. This methodology aimed at taking into account the lack of homogeneity of the degradation usually occurring, as observed for pure films^10^. More details on the physical alteration of the films are present in the SI.

### Historical samples

A set of twenty-seven historical samples of motion picture films were the basis of the developed chemometric models. Their choice was made by the archives: the Phonogrammarchiv of the Austria Academy of Sciences (OEAW, Vienna, Austria), Institut Valencià de Cultura (IVC, Valencia, Spain) and the Deutsches Filminstitut & Filmmuseum (DFF, Frankfurt, Germany). The selection was carefully made to represent the variety of films’ type, dates, brands, state of conservation accessed by AD-strip measurement, and availability to circulate out of the collections due to their intrinsic value. They were molecularly characterized by µFTIR or ATR-FTIR regarding the identification of the plasticizer and the correspondent conservation state accessed by the determination of their DS, with different calibration curves. A detailed study about the equivalence of the DS values obtained by an in depth collected sample analysed in transmission by µFTIR) and a surface analysis on reflection mode (ATR-FTIR) made with the same kind of films is reported elsewhere^18^. The emission of acetic acid was quantified for each entire film reel located inside a standard reference box with a measurement sensor array. Table 2 presents the historical films used in this study and their database archival information.

**Table 2:** Historical films archival information.

File name	Fab. year	AD-strip value	Type	Manufacturing company
Film 01	1935	1.5	BW, sound	Unknown
Film 02	1936	3	BW, positive	AGFA Gevaert
Film 03	1940	1	BW, positive	AGFA Gevaert
Film 04	1940	2.5	BW, positive	AGFA Gevaert
Film 05	1940	2.5	BW, positive	AGFA Gevaert
Film 06	1944	0.5	BW, positive	Voightländer
Film 07	1945	3	BW, sound, positive	AGFA Gevaert
Film 08	1950	0.5	BW, sound, positive	KODAK
Film 09	1950	2.5	BW, sound, positive	AGFA Gevaert
Film 10	1950	3	BW, sound, positive	Perutz Deutschland
Film 11	1953	1	BW, sound, positive	Perutz Nonflam 2
Film 12	1953	2.5	BW, sound, positive	Perutz Nonflam 2
Film 13	1953	3	BW, sound, positive	AGFA Gevaert
Film 14	1953	3	BW, sound, positive	Perutz Nonflam
Film 15	1955	1.5	Color, sound	Mafe Ininf
Film 16	1955	3	BW, sound, positive	Mafe Ininf
Film 17	1959	1	BW, sound	Mafe Ininf
Film 18	1960	0	BW, positive	AGFA Gevaert
Film 19	1962	3	BW, magnetic	AGFA Gevaert
Film 20	1965	3	BW, sound, positive	Ferrania 11
Film 21	1966	3	Color, sound	KODAK
Film 22	1970	3	BW, sound, positive	KODAK
Film 23	1975	2.3	BW, sound, negative	KODAK
Film 24	1978	2	Color, sound	KODAK
Film 25	1989	3	Color, sound, positive	Orwo Deutschland
Film 26	1992	1.5	Color, sound, positive	AGFA Gevaert
Film 27	1998	0.25	Color, positive	Fujicolor

### Free acidity test: AD-strip measurements

AD-strips are paper indicators (1 × 4 cm) of the free acidity inside enclosure storage packages, used by the photographic and cinematographic archives. They were developed exactly for this purpose by the Image Permanence Institute, IPI, in 1997^26^ containing bromocresol green dye and sodium salts indicating through color changes the severity of film degradation. For the measurement, one strip is inserted inside the plastic box or can over the film reel, laying there for two weeks. After that exposure time the change of the initial blue color is analyzed visually by comparing it with a reference pencil with the degradation scale indicated, varying from blue (level 0—not degraded) to yellow (level 3—highly degraded); variations of 0.5 are advised to be considered for a more precise evaluation. According to IPI, at AD level 1.5 degradation reaches the autocatalytic point, when chemical decay continues much faster giving rise to the film’s total destruction. Therefore, this level is crucial and IPI recommends duplication of these films^26^. Currently, AD-strips are produced and commercialized by two companies, IPI and Dancan. The AD-strip measurement of the historical cinematographic films was performed by the archives.

### µFTIR

µFTIR spectra were acquired with a Nicolet Nexus spectrometer coupled to a Continuum microscope (15x) and an MCT-A detector cooled by liquid nitrogen. They were collected in transmission mode from 4000 to 650 cm^−1^ on micro samples compressed with a Thermo diamond anvil cell. 128 scans on each sample were performed in order to improve the signal to noise ratio while fixing the spectral resolution to 8 cm^−1^. Spectra acquisition was performed using Omnic E.S.P. 5.2 software. The degree of substitution was calculated from the obtained at least triplicated IR spectra. The experimental calibration curve used^18^:

1 $$y=0.088 {DS}^{2} - 0.600\mathrm{ DS }+ 1.004,$$

with y being the ratio between the intensity of the OH peak (at 3350 cm^−1^) and that of the C–O–C peak (at 1050 cm^−1^). This approach is based on the fact that the ether bond between the two anhydroglucose rings only suffers variation if chain scission of the polymer occurs, which is not the case considered. It is noteworthy to mention that, in order to eliminate the potential interference of different moisture levels on the height of the OH peak, all samples were conditioned to have the same level of moisture by being placed inside a desiccator with silica gel overnight before performing the measurements. It is important to point out that the degradation of the plasticized films was not homogenous, some regions inevitably degraded faster than others due to the autocatalytic effect of the acetic acid formed, as previously reported^10^. An average over a number of µFTIR spectra was taken over these different-degradation-state regions. The µFTIR method is appropriate when studying artefacts of high historical value since it requires very small sample. Moreover, it provides in-depth/transversal evaluation of the DS of the film.

### ATR-FTIR

ATR-FTIR has the advantage of giving a more averaged value of the DS over the surface, however the penetration depth is a few microns since it is based on the evanescent waves resulting from attenuated total reflected beam. The spectra were obtained by an Agilent Handheld Exoscan 4300 spectrophotometer equipped with a wire-wound source and DTGS detector in the 4500–650 cm^−1^ spectral region. ATR spectra were obtained in reflectance mode with a resolution of 8 cm^−1^ and 64 scans. The degree of substitution was quantified from ATR-FTIR spectra through the ratio $y$ of the O–H peak (3330 cm^−1^) over the C–O–C peak (1030 cm^−1^). Using the experimental calibration curve^18^:

2 $$y=0.039 {DS}^{2} - 0.262\mathrm{ DS }+ 0.427.$$

ATR-FTIR, as an in situ technique was performed for films received as complete reels; others were received as fragments collected from three different parts of the reel (outer, middle and inner parts). These films needed also a faster characterization in order to be sent for determination of their acetic acid emission (Table S3).

### Sensor array for acetic acid vapor quantification

To quantify, in real-time, the amount of acetic acid present in closed containers, an array based on metal oxide semiconducting (MOS) sensors was employed. The array includes two MOS sensors, each constituted by an interdigitated electrode with a layer of a nanostructured metal oxide semiconductor on top, one electrode has a layer of tungsten oxide deposited on it, the other has a layer of tin oxide. Each MOS is heated up to its operating temperature thanks to an integrated heating system localized on the opposite face of the interdigitated electrode. Each MOS responds to exposition to gas with a variation of the resistance measured across the interdigitated circuit. The array system shows a good correspondence between acetic acid concentration, to which the array is exposed, and measured change in resistance^27^.

### Mechanistic model

The mechanistic model (MM) for the CA pure polymer is based on kinetic equations that describe the behavior of the polymer degradation. The complete description and discussion of this model can be found elsewhere^10^. Briefly, two degradation mechanisms were taken into account: the deacetylation reaction under neutral conditions and acid-catalyzed deacetylation. These two degradation channels correspond to the main degradation channels of CA polymer and are described by two parallel kinetic equations^10^. The MM couples density functional theory (DFT) formalism calculations of the activation Gibbs energy ($\Delta {G}^{\ddagger }$) with the transition state theory (TST) to yield the long-term kinetic behavior of the each reaction:

3 $$\frac{{dC}_{A}}{dt}=-\frac{{K}_{B}T}{\mathrm{h}}\mathrm{exp}\left(-\frac{\Delta {G}^{\ddagger }}{RT}\right)\frac{{C}_{A}{C}_{B}}{{C}_{\mathrm{s}}},$$

where ${K}_{B}$, h and R are Boltzmann’s, Plank’s and perfect gas constants respectively, T is the temperature, C_s_ is the standard concentration (usually 1 mol/L), ${C}_{A}$ and ${C}_{B}$ are the concentration of reactants. The MM is a first principles model, that solves, in parallel, as a function of time the following two ordinary differential equations (ODEs). Each ODE corresponds to a considered degradation channel:

4 $$\frac{{dC}_{A}}{dt}=-\frac{{K}_{B}T}{\mathrm{h}}(\mathrm{exp}\left(-\frac{\Delta {G}_{n}^{\ddagger }}{RT}\right)\frac{{C}_{A}{C}_{w}}{{C}_{\mathrm{s}}},$$

5 $$\frac{{dC}_{A}}{dt}=-\frac{{K}_{B}T}{\mathrm{h}}(\mathrm{exp}\left(-\frac{\Delta {G}_{a}^{\ddagger }}{RT}\right)\frac{{C}_{A}{C}_{C}}{{C}_{\mathrm{s}}}.$$

Equation (4) corresponds to the deacetylation under neutral conditions with ${C}_{w}$ is concentration of water and $\Delta {G}_{n}^{\ddagger }$ = 41.61 kcal/mol is the Gibbs free energy of activation obtained from the DFT approach for the deacetylation under neutral conditions. Equation (5) corresponds to the acid-catalyzed deacetylation channel with ${C}_{C}$ is the concentration of acetic acid, responsible for the production of H_3_O^+^ ion, and $\Delta {G}_{a}^{\ddagger }$ = 30.28 kcal/mol is the Gibbs free energy of activation obtained from the DFT approach for the acid-catalyzed deacetylation^10^. The concentrations are calculated in each case as function of time. The concentration of water inside the polymer is expressed as function of *T*, RH and pH. The concentration of AA inside the polymer (sorbed phase) is determined by considering the equilibria with the gas phase at each instant. This is accounted for with the mass balance equation between the polymer and the gas phase. The degradation is determined from the evolution of the number of acetate groups in the CDA polymer and is expressed as percentage of the initial concentration (100% non-degraded). The accuracy of the model was validated through comparison with experimental results of accelerated aging experiments of CDA pure polymer. The ordinary differential equations (Eq. (3)); one for each degradation channel) were solved, as function of time, using the ODE solver, ode45, provided in the software package MATLAB/SIMULINK. The evolution of the concentrations of water and AA were account for at each instant by the solver. The two degradation pathways are considered bimolecular and solved simultaneously.

### Chemometric models and calibration

Multivariate analysis (chemometrics) is a very powerful tool to extract useful information hidden in diverse inputs. The partial least squares (PLS) regression was used in this work. PLS regression is one the most commonly used multivariate analytical techniques, where the model creates linear relations between the inputs and outputs. This method proved to yield satisfactory result in the current study. The Linear regression models were established with MATLAB R2017b (MathWorks, USA) using PLS models, calculated with SIMPLS algorithm with auto-scale pre-processing of the datasets. The Q-residuals were investigated to identify any substantial outliers as well as the Hotelling T-squared to determine the samples affecting mostly the outcome of the model. The model complexity (number of latent variables used) was evaluated based on cross-validation procedure. During the development of the models in this work different input parameters were investigated to inspect the relevance of each one.

Upon building the CM-${k}_{\mathrm{CF}}$, the ${k}_{\mathrm{CF}}$ was determined for a set of films upon fitting the MM output degradation evolution to two points in the lifetime of each film: the initial DS and the current DS, more information are found in the SI. These determined values were then used to train and validate the chemometric model CM-${k}_{\mathrm{CF}}$. The CM-${k}_{\mathrm{CF}}$ model could then be used to calculate ${k}_{\mathrm{CF}}$ for a new film from the fabrication date and the film type (BW, color, sound) as inputs. For training the CM-DS model, FTIR measurements were necessary to provide the DS. However, the application of the model is to predict the DS of a new film from gas data, AD-strip value, and the manufacturing date.

## Results and discussion

### Mechanistic model: applied to plasticized films

The MM model based on differential Eq. (3) was adapted to the case of plasticized films by introducing the ${k}_{\mathrm{CF}}$, as shown in Eq. (6).

6 $$\frac{{dC}_{A}}{dt}=-{k}_{\mathrm{CF}}\frac{{K}_{B}T}{\mathrm{h}}\mathrm{exp}\left(-\frac{\Delta {G}^{\ddagger }}{RT}\right)\frac{{C}_{A}{C}_{B}}{{C}_{\mathrm{s}}}.$$

**Figure 2:**
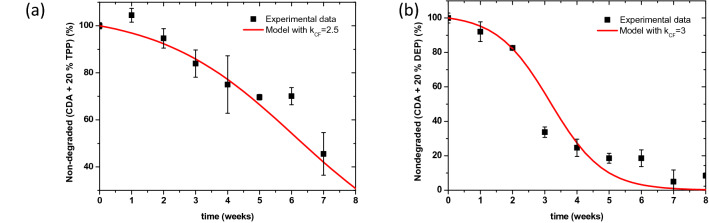
Degradation profiles of plasticized cellulose diacetate polymer. Experimental data are shown in squares along with the corresponding standard deviation error (DS values were obtained using Eq. (1)), while the adapted MM model outcome is represented by the solid red line for the (**a**) 20% weight TPP plasticized polymer, and (**b**) 20% weight DEP plasticized polymer under acidic aging conditions at 70 °C, RH = 80% and initial AA concentration of 1.7899 × 10^–5^ mmol/cm^3^ in the gas phase.

The ${k}_{\mathrm{CF}}$ is an adjustable parameter determined upon obtaining the best fit between the output of the adapted MM and the experimental data from accelerated aging of plasticized films. Accelerated aging experiments were conducted on two types of plasticized CDA polymer, namely CDA + 20%TPP (triphenyl phosphate, weight percentage) and CDA + 20%DEP (diethyl phthalate, weight percentage). This weight percentage was used since it corresponds to a representative percentage in the case of real films^28^. It is important to point that this percentage may differ from historical film to another and also different plasticizer mixtures may be found in historical films^28^. However, this is empirically accounted for by the ${k}_{\mathrm{CF}}$ predicted by the developed chemometric model CM-${k}_{\mathrm{CF}}$. The DS of the aged films with different aging times was determined by µFTIR and expressed in percentage non-degraded with the initial DS (DS of the new film) set as 100% non-degraded. The experimental conditions were used as the storage conditions input parameters (temperature, RH and AA concentration on the gas phase, mass of the polymer, volume of the aging chamber) for the adapted MM (shown in Table 1). Figure 2 shows the output of the adapted MM model in solid red line and the experimental results in symbols along with the associated errors.

In the case of TPP plasticizer (Fig. 2a), the plasticized film was found to degrade 2.5× faster than the pure polymer. In the case of DEP plasticizer, the plasticized film degraded 3× faster than that of a pure polymer. A steep degradation slope was measured between the second and third week of accelerated aging making it to unsuitable to truncate the curve at 40% non-degrade (to focus on the relevant part) due to the scarcity of experimental data in that region. However, it is important to keep in mind that the highest accuracy and relevance of the MM and the adapted MM reside in the slightly degraded region or the region up to the onset of the steep degradation curve. The practical interest of the developed models lies in the slightly degraded regions, since their objective is to predict when the degradation starts to accelerate significantly, in order to decide on preventive actions to take before a total loss of the material. A very good overall agreement is noticed between the adapted MM model outcome and the experimental data. This fundamental study proves the possibility to lump the effect of plasticizer into one parameter (*k*_CF_), however this parameter needs to be determined in the case of historical films.

### Kinetic correction factor determination

**Figure 3:**
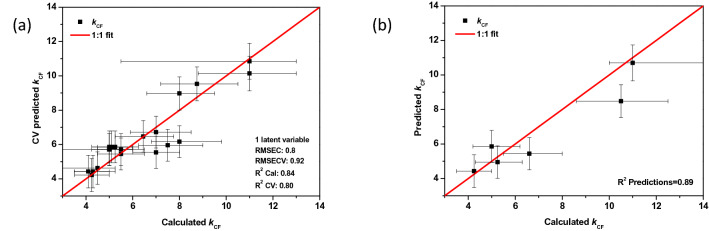
${k}_{CF}$ predictions versus calculated ones. (**a**) Cross validation results, the regression plot between the predicted kinetic correction factor (${k}_{CF}$) along with the root mean square error (RMSE) of cross-validation (vertical bar) versus the ${k}_{CF}$ calculated from the adapted MM along with the corresponding uncertainty (horizontal bar). (**b**) The prediction results of the PLS regression model on an independent dataset or the validation dataset along with the RMSE of prediction (vertical bar) versus the calculated ${k}_{CF}$ determined from adapted MM along with the corresponding uncertainty (horizontal bar). The red line represents the reference line, or the 1:1 fit that corresponds to the perfect case.

To determine how much faster each historical film is degrading compared to the pure polymer, CM-${k}_{\mathrm{CF}}$, a partial least squares (PLS) model to determine the ${k}_{\mathrm{CF}}$ was build. CM-${k}_{\mathrm{CF}}$ considers a dataset of 27 historical films from the three distinct film archives. These films were chosen deliberately, in a representative way taking into account the diversity in fabrication dates and manufacturers as well as the conservation condition assessed by AD-strip values. Upon building CM-${k}_{\mathrm{CF}}$ different input parameters were explored to see the impact of each one on the output. The gas data (rate of release of AA and the maximum concentration of AA), measured using a sensor array (see “Materials and methods” section), proved irrelevant in this model and were discarded. These results can be explained by considering the ${k}_{\mathrm{CF}}$ factor as an intrinsic property of the film, while the gas data are more a product or result of degradation. All gas data used in this work were measured by the same referred sensor array. In the final CM-${k}_{\mathrm{CF}}$ model, the inputs are the film type and year of fabrication, the output is the ${k}_{\mathrm{CF}}$. Upon building the CM-${k}_{\mathrm{CF}}$, the ${k}_{\mathrm{CF}}$ should be provided to train the program. For each film, the ${k}_{\mathrm{CF}}$ is determined upon obtaining the degradation curve that best fits visually the initial and actual DS as shown in Supplementary Fig. S1. The current state of degradation (current DS) was measured by FTIR spectroscopy (ATR-FTIR/µFTIR) and was represented by the actual degree of substitution as non-degraded percentage. The initial degree of substitution was assessed based on the fabrication date and on a critical evaluated relationship with the actual determined DS^18^, detailed in the SI. To obtain the degradation curves, the storage conditions (T, RH, storage box volume) over the lifespan of the films must be provided to the mechanistic model (Table 1). This is a challenging task since no registers of temperature and RH are available for the whole lifespan of each film. Thus, rather estimations that are believed to be the closest to the real conditions (taking into account the film history) were adopted. Effect of uncertainties on each parameter was considered to obtain the confidence limit of the determined ${k}_{\mathrm{CF}}$ values. The temperature was found to be the most critical parameter. Variations of the storage temperature, as well as information about the considered films are found in Supplementary Table S1. For example, considering an average temperature of 20 °C for the storage conditions during the life of a historical film, this temperature value was used in the MM to obtain a kinetic curve for the pure polymer. This kinetic curve was after accelerated by multiplying for a ${k}_{\mathrm{CF}}$ value that best fits the initial and current degradation states. The process was repeated for two more average temperatures, i.e*.* the lower and upper limits of temperature fluctuation, 19 and 21 °C, so that the ${k}_{\mathrm{CF}}$ value that describes the data at each temperature was obtained. This gave the confidence interval for the ${k}_{\mathrm{CF}}$ value arising from the temperature uncertainty. An example of the effect of the obtained ${k}_{\mathrm{CF}}$ values on the kinetic degradation curves is shown in Supplementary Fig. S1. This process was repeated to obtain the confidence interval of the ${k}_{\mathrm{CF}}$ for each film, depending on the corresponding specific storage conditions. Since the actual temperature could be anywhere in between the high temperature and the low temperature limits, this gives an interval for the ${k}_{\mathrm{CF}}$ values instead of just one value for each film. This confidence limit is represented by horizontal error bars in Fig. 3.

Later on, after validation, the developed CM-${k}_{\mathrm{CF}}$ can be used to predict ${k}_{\mathrm{CF}}$ for a new film. The data used to build the CM-${k}_{\mathrm{CF}}$ model is presented in Supplementary Table S2. The CM-${k}_{\mathrm{CF}}$ model yielding the results shown in Fig. 3 was built on 1 latent variable, where it explained roughly 84% of the variance. An increased number of latent variables can lead to an increase in percentage of variance covered (2 LV 89%) however this did not ameliorate the model performance. Moreover, keeping the number of LVs to a minimum can help increasing model robustness. A 21-film calibration dataset was used to establish the mathematical models. Their reliability was evaluated by venetian split cross-validation with a permutation test to avoid overfitting, which is essential in predicting independent samples accurately. The model calibration R-squared is 0.84 and root mean square error of calibration (RMSEC) of 0.8. However, the more realistic determination of error is the root mean square error of cross-validation (RMSECV), which is 0.92. These indicators are standards in evaluating the performance of a multivariant models^29,30^. However, it is important to point out that this evaluation approach has its weaknesses of providing a generalized uncertainty for all the predictions, instead of taking into account the specific uncertainty for each individual prediction^31^. Figure 3b shows a separate set of 6 films that was used as a validation set. Based on the model predictions R-squared, the constructed PLS model gives a prediction accuracy of about 89%.

### Current degree of substitution

**Figure 4:**
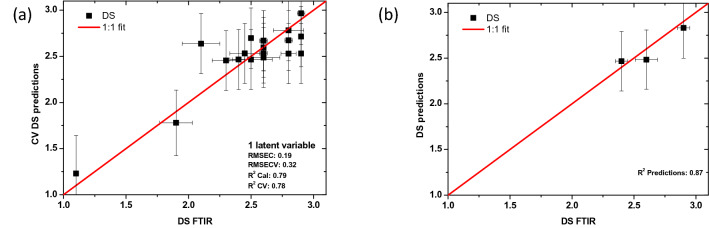
DS predictions versus DS determined from FTIR spectra. (**a**) Cross validation results, the regression plot between the predicted DS along with the (RMSE) of cross-validation (vertical bar) and the DS determined from FTIR spectra along with the corresponding experimental RMSE error (horizontal bar). (**b**) The prediction results of the PLS regression model on an independent dataset or the validation dataset along with the RMSE of prediction (vertical bar) versus the DS determined from FTIR spectra along with the corresponding experimental RMSE error (horizontal bar). The red line represents the reference line, or the 1:1 fit that corresponds to the perfect case.

Determining the current degree of substitution is mandatory since it represents the current state of the film and it serves as the initial DS to predict/simulate the future behavior with the hybrid model. Several studies in the field of conservation have been performed to determine the DS from spectral measurements^17,18,32^ and this approach is well-established. However, in the common practice of the archives, no spectroscopic equipment is available, and all excessive handling of the artefacts is to be avoided. Thus, having a model that gives the DS based on off-gas data is of significant interest. A set of 22 films was studied split into 19 films training dataset and 3 films validation dataset. A multivariate analysis model was built with the DS as an output. Several input parameters were investigated such as fabrication data, AD-strip value, off gas data (gas release rate, maximum concentration), and film type. Unlike the previous chemometric model (CM-${k}_{\mathrm{CF}}$), the film type proved to be of negligible influence on the output and thus it was discarded. The inputs needed for this model are the fabrication date, AD-strip value and off-gas data. In the training and validation process of the model, the DS of the corresponding films must be known. This DS was again evaluated using FTIR spectroscopy (ATR-FTIR/µFTIR). It is important to note that the evaluation of the DS of each film was taken as the average value of the determined DS from several spectra performed at different locations of the films (Supplementary Tables S1, S3). The root-mean-square error associated with each measurement is reported as horizontal bars in Fig. 4.

The importance of each input parameter was analyzed, and the fabrication date along with the gas data proved to be the most important. AD-strip value parameter fell on the edge of the threshold of significance, but it was kept since its value was in-line with the detected gas data, in addition it is a relatively easy parameter to acquire in the archives practice. Thus the CM-DS model was built with 4 inputs namely: fabrication date, AD-strip value, kinetic gas release (ppb/(kg s)) and the maximum gas concentration (ppb/kg). The output of this model is the current DS. The PLS model was built over a dataset of 19 films and a validation set of 3 films. The data for the CM-DS model is presented in Supplementary Table S3. In this model, 1 latent variable covered about 80% of the variance. An increased number of latent variables doesn’t lead to a substantial increase of the percentage of variance covered (2 LV 81%). The model calibration R-squared is 0.79, R-squared CV of 0.78, RMSEC of 0.19 and RMSECV of 0.32. A separate set of 3 films was used as a validation set. As can be seen in Fig. 4b, there is a very good agreement between the predicted and the measured DS, since the line passes through the confidence interval of these points. The predictions R-squared of the constructed PLS is 0.87. This gives good confidence on the ability of CM-DS of determining the DS of a film with unknown DS. A summary of the main features of the developed chemometric models is presented in Table 3.

**Table 3:** A summary of the main features of the developed CM-${k}_{\mathrm{CF}}$ and CM-DS models.

	Training set	Validation set	Latent variables	Variance covered (%)	RMSECV	R^2^ predictions
CM-${k}_{\mathrm{CF}}$	21 films	6 films	1	84	0.92	0.89
CM-DS	19 films	3 films	1	80	0.32	0.87

### Effect of AA adsorbent as function of time

The use of volatile organic compounds adsorbents to clear the atmosphere of corrosive pollutants is a good practice in the preventive conservation processes^19,20^. Some classic adsorbents proved to have good capabilities of adsorbing acetic acid are NaX zeolite in pellet form and the RB4 activated carbon^33^. A more recent study has proposed the use of metal organic frameworks (MOFs) where this new class of materials can be engineered to adsorb acetic acid with high selectivity and efficiency^24^. In the current work, we integrate the effect of the adsorbent on the degradation kinetics through three parameters: the adsorbent amount (${m}_{ad}$), the adsorbent performance through its Henry’s constant (${K}_{{H}_{ad}}$), and the adsorbent replacement time. These parameters can be optimized to maximize the lifetime of the film.

Let us consider a CA film of mass, $m$, stored in a box with free volume, $V$. The box is equipped with an adsorbent of a mass, ${m}_{ad}$. The mass balance equation is then used to determine the AA equilibrium between the gas phase and sorbed phase upon taking into account both the CA film and the AA adsorbent, as follows:

7 $$V\left({C}_{0}-{C}_{out}\right)=m\left({Q}_{in}-{Q}_{0}\right)+{m}_{ad}\left({Q}_{{in}_{ad}}-{Q}_{{0}_{ad}}\right),$$

where, ${C}_{0}$ and ${C}_{\mathrm{out}}$ are the concentration of AA in the atmosphere at t = 0 and time t, respectively. ${Q}_{in}$ is the concentration of AA inside the CA polymer at time t. ${Q}_{0}$ is the total amount of AA produced inside the CA polymer (considering no release of AA; *i.e.* total amount of AA produced in the system from the *t* = 0 to *t*). ${Q}_{{in}_{ad}}$ and ${Q}_{{0}_{ad}}$ are the concentration of AA in the adsorbent at time t and at t = 0, respectively. ${Q}_{{0}_{ad}}$ is considered zero for a new adsorbent, ${Q}_{{in}_{ad}}$ is reset to zero upon changing the adsorbent. From the AA adsorption isotherms on CA, one can relate ${Q}_{in}$ to ${C}_{out}$ via Henry’s law, following the same reasoning in^10^:

8 $${Q}_{in}={K}_{H}\left(p/{p}^{0}\right)={K}_{H}\frac{ZRT}{{p}^{0}} \left(\frac{n}{V}\right)={K}_{H}^{\mathrm{^{\prime}}}{C}_{out},$$

where, ${K}_{H}$ is the temperature independent Henry’s constant^34^, *p* and *p*^0^ are the corresponding pressure and saturation pressure of AA (at temperature *T*), $Z$ is the gas compressibility factor that accounts for the non-ideality of AA in the region of interest, $R$ is the gas constant, ${K}_{H}^{\mathrm{^{\prime}}}$ is the temperature-dependent Henry’s constant of the CA polymer. Moreover, from the AA adsorption isotherms performed on the adsorbent one can relate ${Q}_{{in}_{ad}}$ to ${C}_{out}$ via the temperature-independent or temperature-dependent Henry’s constant ${K}_{{H}_{ad}}$ or ${K}_{{H}_{ad}}^{\mathrm{^{\prime}}}$ respectively.

9 $${Q}_{{in}_{ad}}={{K}_{{H}_{ad}}\left(p/{p}^{0}\right)=K}_{{H}_{ad}}\frac{ZRT}{{p}^{0}} \left(\frac{n}{V}\right)={K}_{{H}_{ad}}^{\mathrm{^{\prime}}}{C}_{out}.$$

Equations (8) and (9) are valid when linear relationships exist between the concentrations of AA in the gas phase and in the sorbed phase. This region corresponds to the Henry’s region or when the AA concentration in the gas phase is very low. It is important to note that the Henry’s region is the region of interest of the current work, since as demonstrated in Supplementary Fig. S2, upon surpassing this region the accelerated degradation starts to become very fast and a total loss of the material becomes inevitable. This means that the objective is to take actions to stay within the Henry’s region. Substituting Eqs. (8) and (9) into Eq. (7) we get:

10 $${C}_{out}=\frac{V{C}_{0}-m{Q}_{0}}{V+m{K}_{H}^{\mathrm{^{\prime}}}+{m}_{ad}{K}_{{H}_{ad}}^{\mathrm{^{\prime}}}}$$

This equation is used to determine the concentration of AA in the atmosphere as function of time and thus the concentration of acetic acid inside the polymer ${Q}_{in}$ at each instant. It is important to recall that ${Q}_{in}$ represents ${C}_{B}$ in Eq. (6) for the acid-catalyzed degradation channel.

### Decision making based on historical film lifetime predictions

**Figure 5:**
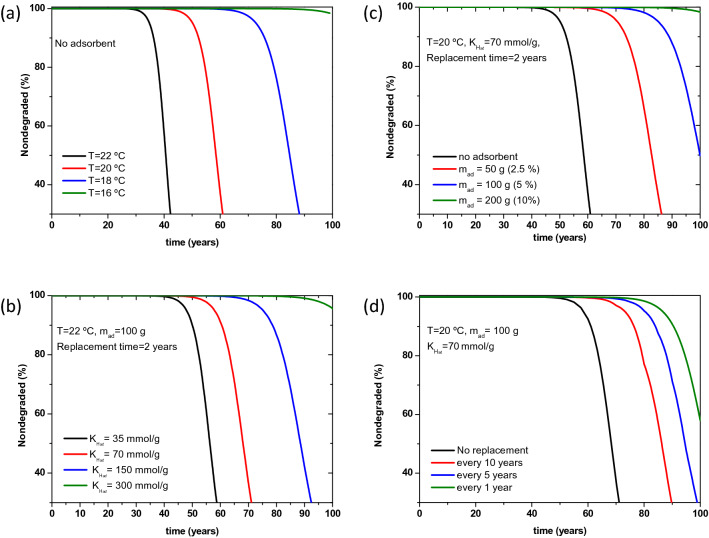
Estimation of the effect of each action on degradation kinetics, upon taking into consideration a typical film of 2 kg that is degrading 7× faster than the pure polymer, stored in a box of 1500 cm^3^ free volume. (**a**) Effect of temperature with no adsorbent present, (**b**) effect of adsorbent properties (through adsorbent Henry’s constant), with fixed T = 20 °C, m_ad_ = 100 g and replacement time = 2 years, (**c**) effect of adsorbent mass (percentage of film mass), with fixed T = 20 °C, adsorbent Henry’s constant = 70 mmol/g and replacement time = 2 years, (**d**) effect of adsorbent replacement time, with fixed T = 20 °C, m_ad_ = 100 g and adsorbent Henry’s constant = 70 mmol/g.

The degradation kinetics proved to be mainly dictated by two factors, the temperature, and the acetic acid concentration inside the film. For this reason, the main tools to combat or slow down degradation kinetics are either freezing or the use of AA adsorbents that can decrease the concentration inside the film. For demonstration purposes a typical film of mass 2 kg, degrading 7× faster than that of the pure polymer model, stored in a box with free volume of 1250 cm^3^ is considered. The effect of temperature is shown in Fig. 5a, where a significant extension of film’s lifetime is seen upon decreasing the storage temperature from ambient temperature of 22 °C to 16 °C. Lowering the temperature to freezing would extend further the film’s lifetime in a non-linear way. However, the freezing solution comes with its inconveniences like energy consumption and additional costs, it may be not even possible in many cases like showrooms. The use of adsorbent might be as effective if suitable adsorbents were used properly.

To simulate the effect of adsorbent, the hybrid model with adsorbent functionality is applied. The Henry’s constant of the CA pure polymer^10^, as well as that of the adsorbent are required. The Henry’s constant of the adsorbent can vary significantly depending on the adsorbent type. While a range of values were reported in literature^24,33^. It is important to point out that these values were estimated from single component AA adsorption. In real application, the adsorbent will be used under moisture conditions, thus, a more accurate assessment is evaluating the Henry’s constant from mixed (AA + water) adsorption isotherms. However, for demonstration purposes several values were considered, and their corresponding effect is reported in Fig. 5b. Figure 5c shows the effect of the amount of adsorbent present in the box. An adsorbent with Henry’s constant of 70 mmol/g, with replacement frequency of 2 years and applied as 10% of the films' mass, can double the film’s lifetime. Figure 5b emphasizes the importance in investing in good quality adsorbent especially when volume constraints are present. The adsorbent replacement time effect is shown in Fig. 5d, a significant amelioration is noticed upon increasing the frequency from once per 10 years to once per year. Further increase in frequency doesn’t lead to a considerable further improvement. It is important to note that the adsorbent effect is accounted for via the Henry’s law, which doesn’t take into consideration the adsorbent saturation and stability. These two parameters become very relevant and need to be considered in long replacement times. Moreover, some adsorbents that proved to be very efficient in AA capture are quite recent and the stability criteria over long periods of time may still not fully established. This point should be addressed carefully before the actual use of adsorbents in close contact with culture heritage items.

An optimization algorithm was used to maximize the lifetime of the film, however it always converged to the set constraints. Meaning that the maximum permitted amount of adsorbent should be used, with the highest available adsorbent Henry’s constant and shortest possible replacement time.

In Fig. 5, we illustrate the effect of several parameters on degradation kinetics for a reference film. Similar scenarios can be simulated for any historical film, and actions can be taken depending on the available resources as well as the conservation objectives.

## Conclusion

A new methodology in evaluating the current degradation state of a historical film along with its evolution over time was successfully implemented. This consisted in a whole package solution for guiding the best actions in preserving CA historical films. The base building block is a first-principles model (MM) for the cellulose acetate pure polymer that is accelerated by a kinetic correction factor (*k*_CF_) to describe the degradation kinetics of real films. A multivariate analysis model (CM-${k}_{\mathrm{CF}}$) is built to predict the correction factor ${k}_{\mathrm{CF}}$ as function of film properties (film type and fabrication date), based on the measured DS of the films as well as the initial DS in a relevant training set of cinematographic films. The developed model provides predictions with accuracy around 89%. The current degradation state, expressed in terms of degree of substitution, is determined via a second chemometric model (CM-DS) with accuracy of predictions of 87%. The model predictions are based on gas data from the array of sensors (the rate of AA emission and the maximum concentration attained) as well as the fabrication date and the AD-strip value; with no need for spectroscopic measurements. The presence of an adsorbent for acetic acid is accounted for in the model, where the effect of different adsorbent parameters (adsorbent amount, Henry’s constant and replacement time) was explored. Evaluating quantitatively the degradation kinetics as function of different storage scenarios enables the user of planning actions accordingly. One of the main factors in the degradation is the storage temperature. When possible lowering down the temperature leads to a significant increase in the lifetime of the artefacts. In the absence of such a possibility, introducing a good adsorbent in adequate amount leads to the same amelioration. The hybrid model solution package is an extremely powerful tool, that integrates the effect of main storage parameters while taking the specificity of each historical film into account. It provides the user with all the needed quantitative information on the effect of each preservation action in the case of each film so that the best decisions can be taken. The proposed study bridges fundamental knowledge with good practice. It gives the user confidence and guidance to take the best actions based on informative predictions, for energy saving and extended conservation time. This study combines first-principles model with data-based models to describe the complex composite material system of historical films. While the proposed approach is quite robust, it is thought of as only the beginning, where upon putting this tool to use in archives a continuous update is necessary. As for the data-based models, upon making more data available more representativity and more accuracy can be achieved.

## Supplementary Information

Supplementary Information.

## Data Availability

Data used to develop the model is disclosed in the Supplementary Data. Additional details may be available to readers upon request to the corresponding authors.

## Abbreviations

AD-strip: Acid detection strip
CDA: Cellulose diacetate
CTA: Cellulose triacetate
CA: Cellulose acetate
AA: Acetic acid
MM: Mechanistic model
DS: Degree of substitution
ATR: Attenuated total reflectance
FTIR: Fourier Transform Infrared spectroscopy
BW: Black and white
TPP: Triphenyl phosphate
DEP: Diethyl phthalate
IPI: Image Permanence Institute
MOS: Metal oxide semiconducting
DFT: Density functional theory
B3LYP: Becke’s three-parameter Lee–Yang–Parr
TST: Transition state theory
ODE: Ordinary differential equations
PLS: Partial least squares
RMSE: Root mean square error
RMSECV: Root mean square error of cross-validation
RMSEC: Root mean square error of calibration
MOF: Metal organic frameworks

## Symbols

T: Temperature
RH: Relative humidity
$k_{{{\text{CF}}}}$: Kinetic correction factor
CM-$k_{{{\text{CF}}}}$: Chemometric model to determine $k_{{{\text{CF}}}}$
CM-DS: Chemometric model to determine DS
$$m_{ad}$$: Adsorbent amount
$K_{{H_{ad} }}$: Adsorbent Henry’s coefficient
$\Delta G^{\ddag }$: Gibbs energy of activation
$C_{A}$ and $C_{B}$: Concentration of reactants
C_s_: Standard concentration
$K_{B}$, h, R: Boltzmann’s, Plank’s, perfect gas constants
$m$: Mass of film
$V$: Box free volume
$C_{0}$: Concentration of AA in the atmosphere at t = 0
$C_{{{\text{out}}}}$: Concentration of AA in the atmosphere at time t
$Q_{in}$: Concentration of AA inside the CA polymer at time t
$Q_{0}$: Total amount of AA produced
$Q_{{in_{ad} }}$: Concentration of AA in the adsorbent at time t
$Q_{{0_{ad} }}$: Concentration of AA in the adsorbent at t = 0
$K_{H}$: CA temperature independent Henry’s constant
*p* and *p*^0^: AA pressure and saturation pressure (at temperature *T*)
$Z$: Gas compressibility factor
$K_{H}^{^{\prime}}$: CA temperature-dependent Henry’s constant
$K_{{H_{ad} }}$: Adsorbent temperature independent Henry’s constant
$K_{{H_{ad} }}^{^{\prime}}$: Adsorbent temperature-dependent Henry’s constant
