# Supplementary material for: Decision making based on hybrid modeling approach applied to cellulose acetate based historical films conservation
Source: Sci Rep. 2021 Aug 9;11:16074. doi: 10.1038/s41598-021-95373-0 (PMC8352898; doi:10.1038/s41598-021-95373-0)
Supplement: Supplementary file 1 — Supplementary Information. [file 41598_2021_95373_MOESM1_ESM.pdf]

Supplementary Information

Decision making based on hybrid modeling approach applied to cellulose acetate based historical films conservation

Abeer Al Mohtar,[1,2]* Moisés L. Pinto,[1,]* Artur Neves,[3] Sofia Nunes,[3] Daniele Zappi[4], Gabriele Varani[4], Ana Maria Ramos,[3] Maria João Melo[3], Nadja Wallaszkovits[5], Juan Ignacio Lahoz Rodrigo[6], Kerstin Herlt[7] and João Lopes[2,]*

[1]CERENA, Departamento de Engenharia Química, Instituto Superior Técnico, Universidade de Lisboa, 1049-001 Lisboa, Portugal. E-mail: abeer.mohtar@tecnico.ulisboa.pt and moises.pinto@tecnico.ulisboa.pt

[2]iMED.ULisboa, Faculdade de Farmácia, Universidade de Lisboa, Av. Prof. Gama Pinto, 1649-003 Lisboa, Portugal. E-mail: jlopes@ff.ulisboa.pt

[3]LAQV-REQUIMTE, Department of Conservation and Restoration and Department of Chemistry, NOVA School of Science and Technology, Universidade NOVA de Lisboa 2829-516 Caparica, Portugal

[4]Biosensor, Via degli Olmetti 44, 00060 Formello (Roma), Italy

[5]Phonogrammarchiv of the Austrian Academy of Sciences, Liebiggasse 5, A-1010 Vienna, Austria

[6] Filmoteca Valenciana, Institut Valencià de Cultura, Plaça de l'Ajuntament 17, 46002 València, Spain

[7]Deutsches Filminstitut & Filmmuseum Schaumainkai 41, 60596 Frankfurt am Main, Germany

Abstract

This document provides supplementary information to “Decision making based on hybrid modeling approach: historical films conservation case study”. Here we present the original degree of substitution data as obtained by µFTIR, the physical appearance of the aged plasticized films, the effect of relative humidity and pH of the medium, the data that was used in the multivariate analysis models, as well as the storage conditions for each film for the calculation of the kinetic correction factor. The reasoning of the determination of the initial degree of substitution is given. The justification on the use of Henry’s law is provided.

Original DS data of plasticized CDA films from the acidic thermal aging and physical appearance

Supplementary Fig. S1 presents the DS as measured by µFTIR from the accelerated aging experiments of the plasticized films. The data shown in Supplementary Fig. S1 yield the experimental data shown in Fig. 1 through DS (%)=DSx100/DS_0, where DS_0 is the initial DS.

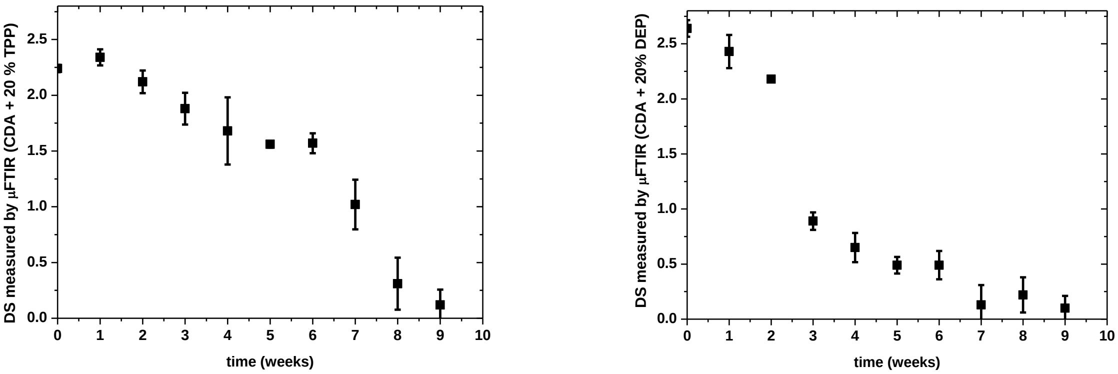

Supplementary Fig. S1. Degradation profiles of plasticized cellulose diacetate polymer. Experimental DS data as obtained from µFTIR spectra through equation (1) along with the corresponding standard deviation error, (a) 20% weight TPP plasticized polymer, and (b) 20% weight DEP plasticized polymer under acidic aging conditions at 70 ˚C, RH=80% and initial AA concentration of $1.7899x10^{-5}$ $mmol/cm^3$ in the gas phase.

Supplementary Fig. S2 shows the freshly fabricated plasticized CA films, where with TPP and DEP plasticizers, the obtained films were transparent with a thickness around 50 µm. These films were artificially aged under acidic conditions.

(a) (b)

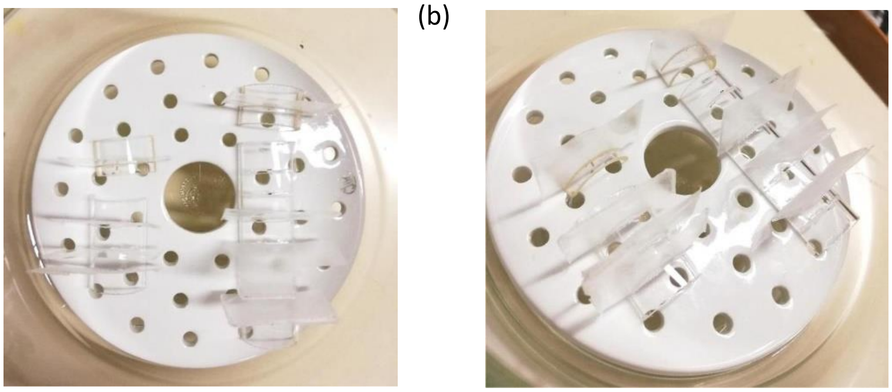

Supplementary Fig. S2. CA plasticized films arranged in glass supports for the acidic ageing experiments, a) samples with TPP plasticizer, b) samples with DEP plasticizer.

After the first week of degradation a large difference was noted in the DEP plasticized films, where a white efflorescence color started to appear in addition to an increase in stiffness. Over the remainder of the ageing time a significant increase in precipitate was noted as can be seen in Supplementary Fig. S3. Unlike the films with DEP, films with TPP didn't show a physical change in the first weeks of the acidic assay.

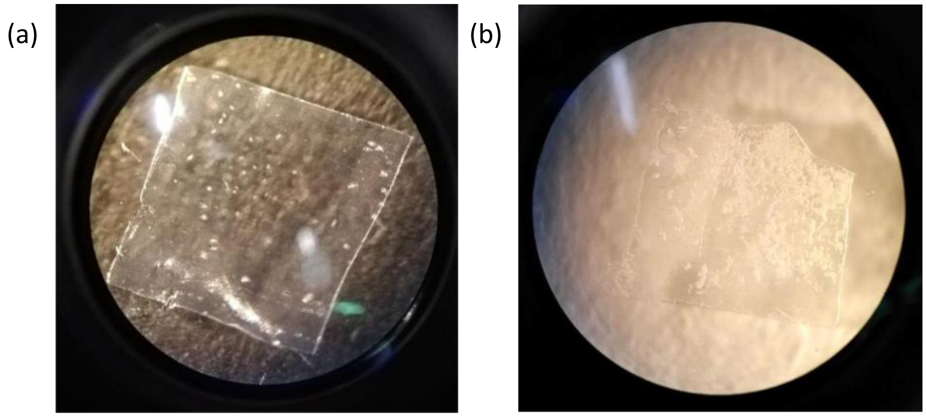

Supplementary Fig. S3. Photograph of CDA samples with DEP a) First week and b) fifth week of degradation.

Effect of relative humidity and medium pH

While it is seen the crucial effect of temperature (Fig 5 (a)) on the degradation kinetics, expressed as an exponential dependence (equation 3). The relative humidity affects the degradation kinetics through influencing the water concentration inside the polymer through[1]:

$$C_{H_2O} = 2.34 + 0.027 \times \mathrm{RH} - 0.007 \times \mathrm{T} + 3.96 \times 10^{11} \mathrm{pH}^{-20.29} \quad \text{(S1)}$$

The increased RH leads to a linear increase in the water concentration inside the polymer. However, it is important to note that this doesn't impact the degradation kinetics significantly as shown in Supplementary Fig. S4 (a). The effect of the acidity of the medium is always taken into account as function of time. As the AA concentration evolves the pH will change according to p$H = -\log\left(\sqrt{Q_{AA} K_a}\right)$, with K_a the dissociation constant equals to 1.8×10^{-5} and Q_{AA} as the concentration of AA inside the film in mol/L. However, in Supplementary Fig. S4 (b) the value of pH was manually fixed to a certain level to demonstrate its effect. The nonlinear dependence of the degradation kinetics on the pH is manifested. It is important to note that as degradation advances, and the concentration of AA increases, this not only gives rise to the second degradation channel (acid-catalyzed degradation channel with much lower activation energy) but also it leads to water retention in an exponential way.

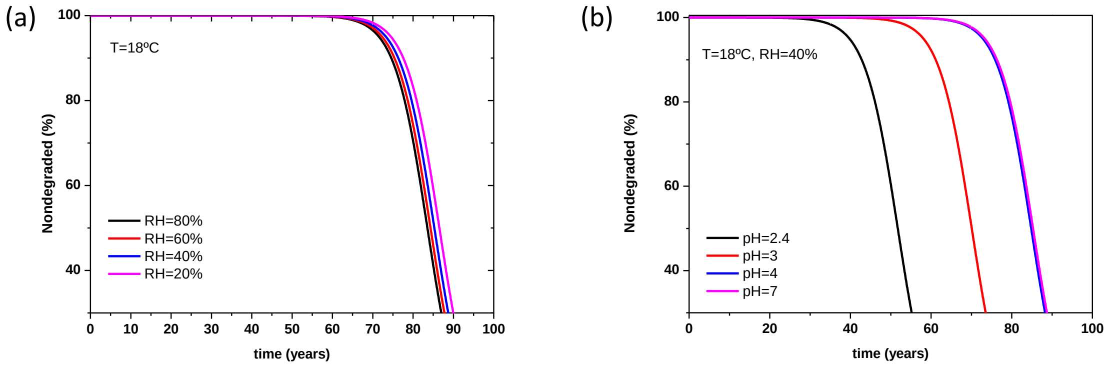

Supplementary Fig. S4. Estimation of the effect of (a) RH and (b) pH on degradation kinetics, upon

taking into consideration a typical film of 2 kg that is degrading 7x faster than the pure polymer, stored at T=18 ºC in a box of 1500 cm^3 free volume with no initial AA concentration.

Data for CM-k_{CF}

A demonstration on the effect of k_{CF} on the degradation kinetics is presented in Supplementary Fig. S5. Where a typical film storage conditions were plugged into the MM and the outcome is adapted to obtain the best fit. The search for the k_{CF} confidence limit consisted of conducting this procedure twice for the low and high temperature limits. The storage conditions for each film used in the CM-k_{CF} dataset are shown in Supplementary Table S1 along with the corresponding k_{CF}. The training and validation datasets X-block and Y-block used to build CM-k_{CF} are shown in Supplementary Table S2.

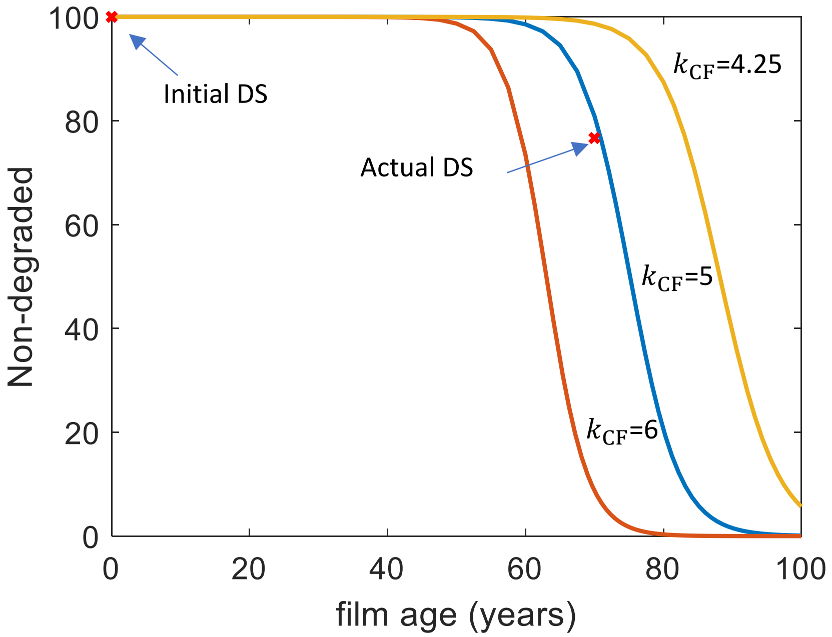

Supplementary Fig. S5. Example of k_{CF} determination for film label 90, the k_{CF} interval in this case [4.25, 6] corresponding to temperature interval 20 ± 1°C, with 5 as the value corresponding to the average temperature (20 ºC). Note that in this figure all curves are simulated at T=20 ºC the only changing parameter is the k_{CF} to illustrate its impact on the degradation kinetics.

Supplementary Table S1. Data used for the calculation of the k_{CF} values for the films used in CM-k_{CF}.

**Film name**	Fab. Year	RH (%)	Box volume (cm^3)	m (g)	Initial DS	Actual DS	High T	Low T	T-average	High T k_{CF}	Low T k_{CF}	k_{CF} fed to CM k_{CF}
Film 01	1935	70	2453	2200	3	2.9	17	19	18	4.3	6.3	5.25
Film 02	1936	40	445	2000	2.5	2.4	21	19	20	3.5	5	4.25
Film 03	1940	40	3598	2000	2.7	2.5	21	19	20	3.5	5	4.25
Film 04	1940	40	420	500	2.7	2.6	21	19	20	3.5	5	4.1
Film 05	1940	40	2398	1500	3	2.5	21	19	20	5.25	3.5	4.3
Film 06	1944	40	700	2300	2.7	2.6	24	19	20	2.1	5.25	4.5
Film 07	1945	40	2189	1500	2.5	1.1	21	19	20	6.5	4.5	5.5
Film 08	1950	40	2148	900	2.5	1.9	21	19	20	4.5	6.5	5.5
Film 09	1950	40	553	900	2.7	2.5	24	19	20	2.4	6	5
Film 10	1950	40	1998	2000	3	2.3	21	19	20	4.25	6	5
Film 11	1953	40	971	3600	3	2.3	21	19	20	4.5	6.25	5.25
Film 12	1953	40	972	3400	3	2.6	21	19	20	4.2	6.2	5
Film 13	1953	40	1155	1900	3	2.6	21	19	20	4.25	6	5
Film 14	1953	40	971	3600	3	2.6	21	19	20	4.25	6	5.1
Film 15	1955	70	4148	4100	3	2.1	17	19	18	6.6	9.5	8
Film 16	1955	70	2453	2100	3	2.6	17	19	18	6.25	9	7.5
Film 17	1959	70	2378	1500	3	2.5	17	19	18	6.8	9.8	8
Film 18	1960	50	961	1100	3	2.75	20	18	19	5.5	8	6.6
Film 19	1962	40	1794	2000	3	0.75	21	19	20	6	8	7
Film 20	1965	40	1348	3100	3	2.4	21	19	20	5.25	7.75	6.45
Film 21	1966	70	4148	3200	3	2.8	17	19	18	7.2	10.5	8.75
Film 22	1970	40	1249	2400	3	2.45	21	19	20	5.9	8.5	7
Film 23	1975	70	4148	3400	3	2.8	17	19	18	8.6	12.5	10.5
Film 24	1978	70	4148	3200	3	2.9	17	19	18	8.8	13	11
Film 25	1989	40	1848	2000	3	2.6	21	19	20	10	14.5	11
Film 26	1992	70	386	3200	3	2.9	24	19	20	5.5	13	11
Film 27	1998	70	386	3000	3	2.9	24	19	20	6.75	17	14.5

Supplementary Table S2. Dataset input (X-block) and output (Y-block) used to train and validate CM-k_{CF}.

	**X-BLOCK**				**Y-BLOCK**
File name	Fab. Year	BW	Sound	Color	k_{CF} values
Film 01	1935	1	1	0	5.25
Film 02	1936	1	0	0	4.25
Film 03	1940	1	0	0	4.1
Film 04	1940	1	0	0	4.25
Film 05	1940	1	0	0	4.3
Film 06	1944	1	0	0	4.5
Film 07	1945	1	1	0	5.5
Film 08	1950	1	1	0	5
Film 09	1950	1	1	0	5
Film 10	1950	1	1	0	5.5
Film 11	1953	1	1	0	5
Film 12	1953	1	1	0	5
Film 13	1953	1	1	0	5.1
Film 14	1953	1	1	0	5.25
Film 15	1955	0	1	1	8
Film 16	1955	1	1	0	7.5
Film 17	1959	1	1	0	8
Film 18	1960	1	0	0	6.6
Film 19	1962	1	0	0	7
Film 20	1965	1	1	0	6.45
Film 21	1966	0	1	1	8.75
Film 22	1970	1	1	0	7
Film 23	1975	1	1	1	10.5
Film 24	1978	0	1	1	11
Film 25	1989	0	1	1	11
Film 26	1992	0	1	1	11
Film 27	1998	0	0	1	14.5

Data for CM-DS

The training and validation datasets used to build CM-DS are shown in Supplementary Table S3.

Supplementary Table S3. Dataset input (X-block) and output (Y-block) used to train and validate CM-DS.

	X-block				Y-block
File name	Fab. Year	AD_strip value	Rate (ppb/kg.s)	Max. Concentration (ppb/kg)	DS-FTIR
Film 01	1935	1.5	0.1635	2.19E+03	DS 2.9 ± 0.02 (µFTIR, 3 spectra)
Film 03	1940	1	0.428	2093	2.6 ± 0.01 (FTIR-ATR, 5 spectra)
Film 05	1940	2.5	0.226666667	3733.087773	DS 2.5 ± 0.17 (µFTIR, 6 spectra)
Film 06	1944	0.5	0.0711	104.6739	2.6 ± 0.03 (µFTIR, 3 spectra)
Film 07	1945	3	469.82	104857.0806	DS 1.1 ± 0.02 (µFTIR, 3 spectra)
Film 08	1950	0.5	0.000222222	3.777777778	DS 2.5 ± 0.05 (µFTIR, 2 spectra)
Film 09	1950	2.5	81.98888889	146269.5	DS 1.9 ± 0.13 (µFTIR, 4 spectra)
Film 11	1953	1	1.398805556	468.6666667	DS 2.6 ± 0.03 (FTIR-ATR, 5 spectra)
Film 12	1953	2.5	1.078368421	2745.210526	DS 2.6 ± 0.03 (FTIR-ATR, 5 spectra)
Film 13	1953	3	4.578611111	10248.67222	DS 2.3 ± 0.11 (FTIR-ATR, 5 spectra)
Film 14	1953	3	3.516176471	3848.786491	DS 2.6 ± 0.09 (FTIR-ATR, 9 spectra)
Film 15	1955	1.5	0.839365854	624.3902439	2.1 ± 0.15 (FTIR-ATR, 5 spectra)
Film 16	1955	3	2.831380952	5705.238095	2.6 ± 0.13 (FTIR-ATR, 5 spectra)
Film 17	1959	1	2.461933333	398.58	2.5 ± 0.01 (FTIR-ATR, 3 spectra)
Film 18	1960	0	0.136909091	617.5181818	2.8 ± 0.12 (µFTIR, 3 spectra)
Film 20	1965	3	26.48096774	13370.20368	DS 2.4 ± 0.05 (FTIR-ATR, 5 spectra)
Film 21	1966	3	6.5259375	7488.4375	DS 2.8 ± 0.06 (µFTIR, 3 spectra)
Film 22	1970	3	4.520833333	12513.44	2.5 ± 0.12 (µFTIR, 4 spectra)
Film 23	1975	2.3	0.632441176	304.2647059	DS 2.8 ± 0.03 (µFTIR, 3 spectra)
Film 24	1978	2	0.13196875	336.90625	DS 2.9 ± 0.01 (µFTIR, 3 spectra)
Film 26	1992	1.5	2.19E-05	0.608926938	2.9± 0.03 (µFTIR, 3 spectra)
Film 27	1998	0.25	0	0.00	2.9 ± 0.03 (µFTIR, 3 spectra)

Justification on initial DS estimates.

Each film analyzed has its own historical background, having been produced in different industrial contexts that are unknown to us. As such, it is impossible to predict with confidence which DS was used in the production of these films. A rough estimation was made based on the current DS of the films and on manufacture information obtained from the literature. Kodak, Defender, Ansco, Dupond, Agfa and Gevaert produced cellulose diacetate motion picture film from 1922 to 1955. Although companies such as Kodak and Gavaert developed the cellulose triacetate base during the 1930s, it was only used in motion picture film after 1948. In the period between 1948 and 1955 both diacetate and triacetate coexisted in the market. Based on the literature, cellulose diacetate acetyl content (%) would range from 38.7 to 40.1 corresponding to a DS 2.3 to 2.5 and cellulose triacetate acetyl content (%) would range from 43.7 to 44.8 corresponding to a DS 2.9 to 3.0[2]. Knowing this, a rough estimative of the NEMOSINE samples manufacturing (initial) DS can be made:

1) samples dated prior to 1948 have a diacetate base and a manufacturing DS 2.5;

2) samples dated from 1955 onward have a triacetate base and a manufacturing DS 3.

3) samples which dating falls in the period between 1948-1955 need a more careful examination. Comparing the calculated DS with the conservation condition of the sample it is possible to propose a possible manufacturing DS value. The justification for these samples is described in Supplementary Table S4.

4) Samples film 03, film 04 and film 06 did not follow presupposition 1). According to Fengel and Wegener[3], cellulose acetate for films could have a manufacturing DS between 2.2 and 2.7 (acetyl content 36.5-42-2%), Supplementary Table S5 . The higher value of 2.7 was used as a rough estimate for the samples dated prior to 1948 having a DS higher than 2.5. The justification for these samples is described in Supplementary Table S4.

Supplementary Table S4. Initial DS estimates and justifications for samples dated between 1948-1955.

Sample	Manufacturer	Date	AD-Strip	DS	Initial DS estimate	Estimate justification
Film 03	AGFA	1936-45	N.A. (film is in good condition)	2.6	2.7	Dated prior to 1948 and a higher DS than 2.5. The film condition is good which correlates with the difference of 0.1 between the initial and the actual DS
Film 04	AGFA	ca. 1940	2.5	2.5	2.7	Dated prior to 1948 and a DS 2.5. The poor-critical film condition diagnosed by the AD-strip level does not correlate with a DS 2.5. This film is from the same period and manufacturer of film 95.
Film 05	AGFA	1930-50	2.5	2.5	3	A decrease of an initial DS 3 to 2.5 correlates with the poor- critical film condition diagnosed by the AD-Strip level. Thus, the film should be from the late 40s.
Film 06	Voigtländer	1943-44	N.A.	2.6	2.7	Dated prior to 1948 and a higher DS than 2.5.
Film 07	Gevaert	1940-50	3	1.1	2.5	A decrease of an initial DS 2.5 to 1.1 correlates with the critical film condition diagnosed by the AD-strip level.
Film 09	Gevaert	1950-60	2.5	1.9	2.5	A decrease of an initial DS 2.5 to 1.9 correlates with the poor-critical film condition diagnosed by the AD-Strip level.

Film 10	Perutz	1950	3	2.3	3	A decrease of an initial DS 3 to 2.3 correlates with the critical film condition critical by the AD-Strip level
Film 12	Perutz	1953	2.5	2.6	3	A decrease of an initial DS 3 to 2.3 correlates with the poor critical film condition diagnosed by AD-strip level.
Film 13	AGFA	1950-53	3	2.3	3	A decrease of an initial DS 3 to 2.3 correlates with the critical film condition diagnosed by AD-strip level.

Supplementary Table S5. Table by Wengel and Wegener (2011), entitled: Various types of cellulose acetate [3].

Acetyl content (%)	Degree of substitution	Common solvents	Application
13.0-18.6	0.6-0.9	Water	
22.2-32.2	1.2-1.8	2-Methoxy ethanol	Plastics, lacquers
36.5-42.2	2.2-2.7	Acetone	Fibres, photographic films
43.0-44.8	2.8-3.0	Chloroform	Fabrics, foils, fibres

Justification on the use of Henry's law

Upon relating the concentration of acetic acid in the sorbed phase to that in the gas phase either in the case of CA pure polymer[1] or in the case of adsorbents[4,5], a linear relation is often observed at least up to 0.06 p/p^0, where p is the pressure of AA in the atmosphere and p^0 is the saturation pressure of AA at ambient temperature. This partial pressure, at the experimental temperature of 25 ˚C, corresponds to a pressure of around 1000 mTorr. One of the hybrid model outcomes is the concentration of AA in the atmosphere in mmol/cm^3. We use the non-ideal gas law presented in equation (6), to convert the concentration into AA pressure. Supplementary Fig. S6 shows that the Henry's region is surpassed only when the accelerated degradation kinetics kicks off. It is important to note that, the interest of the developed hybrid model lies in the region before the onset of the accelerated degradation and the actions to be taken to stay in that region.

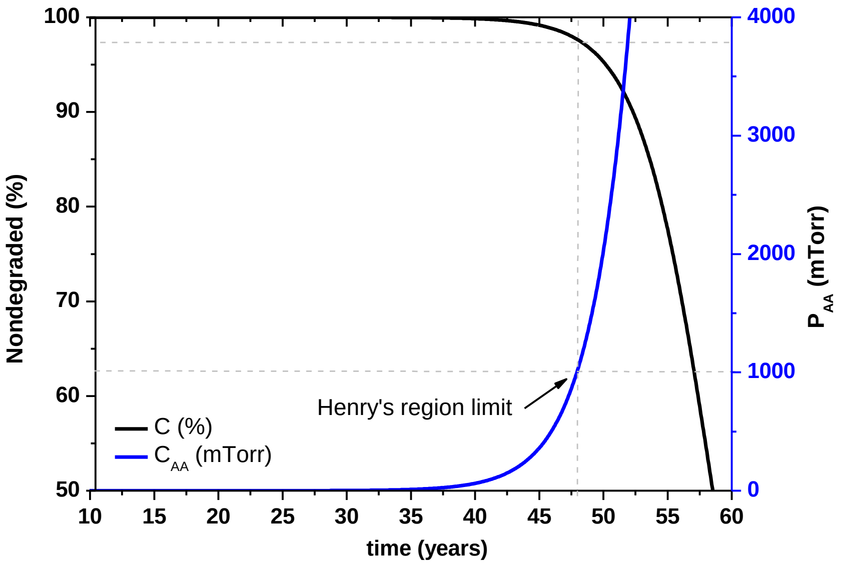

Fig. S6. The degradation kinetics along with the AA emission upon considering a typical film of 2 kg that is degrading 7x faster than the pure polymer, stored in a box of 1500 cm^3 free volume at T=20 ˚C.

References

(1) Al Mohtar, A.; Nunes, S.; Silva, J.; Ramos, A. M.; Lopes, J.; Pinto, M. L. First-Principles Model to Evaluate Quantitatively the Long-Life Behavior of Cellulose Acetate Polymers. *ACS Omega* **2021**, *6* (12), 8028–8037. https://doi.org/10.1021/acsomega.0c05438.

(2) Roldão, É. A Contribution for the Preservation of Cellulose Esters Black and White Negatives. PhD Thesis, NOVA School of Science and Technology, Universidade NOVA de Lisboa, 2018. https://run.unl.pt/handle/10362/59914

(3) Fengel, Dietrich, and Gerd Wegener. *Wood: Chemistry, Ultrastructure, Reactions*; Walter de Gruyter, 2011.

(4) Cruz, A. J.; Pires, J.; Carvalho, A. P.; De Carvalho, M. B. Adsorption of Acetic Acid by Activated Carbons, Zeolites, and Other Adsorbent Materials Related with the Preventive Conservation of Lead Objects in Museum Showcases. *J. Chem. Eng. Data* **2004**, *49* (3), 725–731. https://doi.org/10.1021/je034273w.

(5) Dedecker, K.; Pillai, R. S.; Nouar, F.; Pires, J.; Steunou, N.; Dumas, E.; Maurin, G.; Serre, C.; Pinto, M. L. Metal-Organic Frameworks for Cultural Heritage Preservation: The Case of Acetic Acid Removal. *ACS Appl. Mater. Interfaces* **2018**, *10* (16), 13886–13894. https://doi.org/10.1021/acsami.8b02930.
